# Porcine deltacoronavirus nonstructural protein 2 inhibits type I and III IFN production by targeting STING for degradation

**DOI:** 10.1186/s13567-024-01330-w

**Published:** 2024-06-17

**Authors:** Xiqian Liu, Likai Ji, Yuqiang Cheng, Linghe Kong, Songhua Xie, Juan Yang, Jiaqi Chen, Zhaofei Wang, Jingjiao Ma, Hengan Wang, Yaxian Yan, Jianhe Sun

**Affiliations:** 1 Shanghai Key Laboratory of Veterinary Biotechnology, School of Agriculture and Biology, Shanghai Jiao Tong University, Shanghai, China; 2 School of Medicine, Jiangsu University, Zhenjiang, Jiangsu China

**Keywords:** Porcine deltacoronavirus, nonstructural protein 2, STING, interferon production

## Abstract

Porcine deltacoronavirus (PDCoV) is an enteropathogenic coronavirus that has been reported to use various strategies to counter the host antiviral innate immune response. The cGAS-STING signalling pathway plays an important role in antiviral innate immunity. However, it remains unclear whether PDCoV achieves immune evasion by regulating the cGAS-STING pathway. Here, we demonstrated that the nonstructural protein 2 (nsp2) encoded by PDCoV inhibits cGAS-STING-mediated type I and III interferon (IFN) responses via the regulation of porcine STING (pSTING) stability. Mechanistically, ectopically expressed PDCoV nsp2 was found to interact with the N-terminal region of pSTING. Consequently, pSTING was degraded through K48-linked ubiquitination and the proteasomal pathway, leading to the disruption of cGAS-STING signalling. Furthermore, K150 and K236 of pSTING were identified as crucial residues for nsp2-mediated ubiquitination and degradation. In summary, our findings provide a basis for elucidating the immune evasion mechanism of PDCoV and will contribute to the development of targets for anti-coronavirus drugs.

**Supplementary Information:**

The online version contains supplementary material available at 10.1186/s13567-024-01330-w.

## Introduction

PDCoV belongs to the deltacoronavirus genus of the *Coronaviridae* family and was first detected in Hong Kong in 2012 [1]. In February 2014, outbreaks of PDCoV infection in piglets were first reported in the United States, followed by subsequent occurrences in China, Korea, Thailand, and numerous other countries [2]. Infected piglets typically experience watery diarrhoea, vomiting, and dehydration, with a mortality rate of nearly 100% in neonatal piglets, resulting in considerable economic losses for the global pig industry [3]. Additionally, one study reported the presence of PDCoV viral particles in plasma samples from three Haitian children, confirming the potential risk of cross-species transmission and the public health hazards associated with PDCoV infection [4]. PDCoV is currently known as the smallest single-stranded positive-sense RNA coronavirus, with a length of approximately 25.4 kb. The genome of PDCoV contains 15 nonstructural proteins (nsp2-16), four structural proteins, namely, the spike (S), envelope (E), membrane (M), and nucleocapsid (N) proteins, and three accessory proteins (NS6, NS7, and NS7a) [5]. The nonstructural proteins are essential for viral transcription, replication, protein post-translational degradation, and host modification, particularly for the regulation of host innate immunity [6, 7]. However, the functions of some nonstructural proteins, such as nsp2, remain unknown.

The interferon response, which includes type I IFNs (IFNα and IFNβ) and type III IFNs (IFNλ), is one of the first and most crucial components of the host innate immune system against viral infections [8]. Type I IFN is commonly regarded as the first-line immune defence, generally triggering a broad and systemic antiviral response, while type III IFN is acknowledged as a critical local innate defence against pathogens invading mucosal barriers, such as the intestinal and respiratory barriers [9]. Moreover, type I and III IFNs exhibit apparent similarities in intracellular signalling events and subsequent expression of interferon-stimulated genes (ISGs) [8]. Therefore, it is equally important to explore the role of both type I and III IFNs in host innate antiviral immunity against PDCoV infection. Previous studies have shown that PDCoV infection can inhibit IFN production in porcine cells, thus drawing significant attention to the immune evolution mechanism of PDCoV [10, 11].

RNA-stimulated signalling is mainly dependent on the adaptor protein mitochondrial antiviral signalling (MAVS), whereas the signalling triggered by DNA mainly relies on the adaptor protein stimulator of interferon genes (STING) during viral infection [12]. Due to the crosstalk between RNA and DNA sensing pathways, the activation of cGAS-STING signalling by RNA viruses has gradually been discovered [13, 14]. SARS-CoV-2 infection was reported to activate cGAS-STING through DNA damage, chromatin damage, and mitochondrial DNA release [15–17]. Although there is currently no known evidence that PDCoV activates the cGAS-STING pathway, similar mechanisms are likely to exist based on relevant studies of other coronaviruses [15–18]. RNA viruses have evolved immune evasion strategies that antagonize not only the retinoic acid-induced gene I-like receptor (RLR) signalling pathway but also the cGAS-STING signalling pathway to ensure their survival [19]. For instance, hepatitis C virus NS4B suppresses type I IFN by impairing the interaction between STING and TBK1 [20]. SARS-CoV-2 ORF10 inhibits cGAS-STING signalling by blocking the translocation of STING [21]. In addition, the Tembusu virus NS2B3 protease blocks IFNβ induction by cleaving STING [22]. However, whether PDCoV targets cGAS-STING signalling for immune evasion is unknown.

A critical strategy for the successful persistence of coronavirus infection is to evade the host’s innate immune response. On the one hand, viruses can hijack host proteins or noncoding RNAs to achieve immune evasion [23]. On the other hand, viruses can also inhibit the innate immune response through their own virally encoded proteins. Several viral proteins encoded by PDCoV, such as N, NS6, nsp5, nsp10, and nsp15, have been identified as antagonists of the RLR-mediated type I IFN response [24–28]. Our previous reports revealed that PDCoV N suppresses both key host proteins upstream and downstream of RLR signalling, leading to a decrease in porcine IFNβ [29, 30]. One previous study revealed that PDCoV suppresses IFN-λ1 production in porcine intestinal mucosal epithelial cells (IPI-2I cells) [10]. However, there is limited knowledge regarding the underlying mechanism involved. In deltacoronavirus, there is no orthologue of nsp1, which is present in alphacoronaviruses and betacoronaviruses. PDCoV nsp2 is the initial N-terminal cleavage product of the polyproteins and exhibits the most divergent conservation among the four different genera; however, limited studies have been conducted on this protein. Based on structural biology and interactome research, SARS-CoV-2 nsp2 is predicted to be essential for viral transcription and translation initiation [31]. Transmissible gastroenteritis virus (TGEV) nsp2 has been reported to be involved in the regulation of inflammation [32]. However, additional functions of coronavirus nsp2, especially in antagonizing the IFN response, have remained unclear. Here, we report for the first time that the PDCoV-encoded nsp2 could antagonize the cGAS-STING signalling pathway. Mechanistically, PDCoV nsp2 directly targeted pSTING for ubiquitin-mediated degradation, resulting in the inhibition of cGAS-STING-induced type I and III IFN production. These findings provide novel insight into host‒virus interactions during PDCoV infection.

## Materials and methods

### Cells, viruses, and reagents

HEK293T, LLC-PK1, and IPEC-J2 cells were purchased from the American Type Culture Collection (ATCC). HEK293T (bio-72947) is an epithelial cell line derived from human kidney cells. LLC-PK1 (bio-131297) is an epithelial cell line derived from porcine kidney cells. IPEC-J2 (bio-131297) is an epithelial cell line derived from the porcine small intestine. These cells were maintained in Dulbecco’s modified Eagle’s medium (DMEM) supplemented with 10% foetal bovine serum (Gibco, USA) at 37 °C in 5% CO_2_. The PDCoV-CHSH-2016 strain was isolated, identified, and preserved in our laboratory [30]. The recombinant vesicular stomatitis virus (VSV-GFP) has been described in a previous study [29]. A rabbit anti-STING polyclonal antibody was purchased from ABclonal (Wuhan, China). Rabbit anti-TBK1 and anti-IRF3 polyclonal antibodies were purchased from CST (USA). Mouse monoclonal antibodies against Flag, HA, Myc, and glyceraldehyde-3-phosphate dehydrogenase (GAPDH) were purchased from Abmart (Shanghai, China). Rabbit polyclonal antibodies against Flag, HA, and Myc were purchased from Yeasen (Shanghai, China). Alexa Fluor 488-conjugated goat anti-rabbit and 647-conjugated goat anti-mouse antibodies were purchased from Beyotime (Shanghai, China). A mouse monoclonal antibody against the PDCoV N protein was generated in our laboratory as described previously [29]. MG132 was obtained from Selleck (USA). Poly(dA:dT) was obtained from InvivoGen (USA).

### Plasmids

The porcine *STING* (*pSTING*), porcine *cGAS* (*pcGAS*), and various PDCoV genes were amplified from cDNA generated from LLC-PK1 cells or PDCoV-positive samples and then cloned and inserted into plasmids through standard molecular biology techniques. Several mutations of *pSTING* were cloned and inserted into the pcDNA3.1-Flag plasmid, including truncated amino acid regions (1–149, 1–195, 1–343, and 1–379) and mutations in which lysines were mutated to arginines (K61R, K112R, K150R, K187R, K236R, K289R, K150/236R). PDCoV nsp2 truncations were cloned and inserted into the plasmid pCMV-Myc, including truncated regions (1–189, 1–303, 1–357, and 1–476). The PCR primers used are listed in Table 1, and all the constructs were confirmed by DNA sequencing. The porcine *TBK1* (*pTBK1*), porcine *IKKε* (*pIKKε*), porcine *IRF3-5D* (*pIRF3-5D*), porcine *IRF7* (*pIRF7*), PDCoV E, M, S, and N, and porcine IFNβ (pIFNβ), porcine ISRE (pISRE), and porcine NF-Κb (pNF-κB) promoter reporter plasmids were constructed in our laboratory as described previously [29]. The porcine IFNλ1 (pIFNλ1) promoter was amplified from genomic DNA extracted from LLC-PK1 cells. Ubiquitin (Ub) and Ub mutants, including K48 and K63, have been described previously [29].

**Table 1:** **Primer sequences used for constructing the expression plasmids**

Primers	Sequence (5′ → 3′)
pSTING-PF	CTAGCGTTTAAACTTAAGCTTATGCCCTACTCCAGCCTGCA
pSTING-PR	GTCCTTGTAATCCATGCGGCCGCAGAAGATATCTGAGCGGAGTGGAA
pcGAS-PF	CCAGATTACGCTTCGGGTACCATGGCGGCCCGGCGGGGA
pcGAS-PR	GCCCTCTAGACTCGAGCGGCCGCCTACCAAAAAACTGGAAATCCATTG
pSTING-K61R-PF	TAGGACTGCTGGTGCGGGGGCTCTGCAGTCTGGCG
pSTING-K61R-PR	CCGCACCAGCAGTCCTATCTGCTGGGAGGCCA
pSTING-K112R-PF	TCTCCATCCGAGACCGGGCTGGCCTGCCCCTCCCC
pSTING-K112R-PR	CCGGTCTCGGATGGAGAAGTAGAAGTAGCAGG
pSTING-K150R-PF	CTGTGAACGGAGGAACTTCAACGTGGCTCATG
pSTING-K150R-PR	AGTTCCTCCGTTCACAGATTGCAGAGACTTCAGC
pSTING-K187R-PF	AATCAGCGCCACCGGAACGTACTCGGGGGCATAGG
pSTING-K187R-PR	TTCCGGTGGCGCTGATTATAAGCTTGGATCCG
pSTING-K236R-PF	ATCCGGGGCCGGGTGTACACCAACAGCATCTA
pSTING-K236R-PR	TACACCCGGCCCCGGATGCCAGCACGGTCGGC
pSTING-K289R-PF	GCAGGCCCGGCTCTTCTGCCGGACCCTCGAAG
pSTING-K289R-PR	AGAAGAGCCGGGCCTGCTCGAGCCGATCCTCC

### Dual-luciferase reporter assays

HEK293T or LLC-PK1 cells were grown in 24-well plates and co-transfected with the indicated plasmids. After 24 h, the cells were harvested and lysed in lysis buffer, and the luciferase activities of pIFNβ, pIFNλ1, pISRE, pNF-κB, and the TK-Renilla reporter were measured with a Dual-Luciferase Reporter Assay System (Promega, USA) according to the manufacturer’s instructions. The data are shown as the relative firefly luciferase activities normalized to the Renilla luciferase activities from three independently conducted experiments. The relative luciferase activity was relative to that of an empty vector control.

### Coimmunoprecipitation (Co-IP) and Western blotting

HEK293T or LLC-PK1 cells were co-transfected with an empty vector or the recombinant expression plasmid. At 28 h post-transfection, the cells were harvested by adding lysis buffer (50 mM Tris–HCl (pH 7.4), 150 mM NaCl, 1% NP-40, 10% glycerin, 0.1% sodium sulfate, and 2 mM Na_2_EDTA) containing a protease inhibitor mixture plus the protease inhibitor phenylmethylsulfonyl fluoride (PMSF). The cell lysates were then immunoprecipitated at 4 °C with mouse anti-Flag or anti-Myc magnetic beads (Selleck, USA). The immunoprecipitates were washed four times with 1 × Tris-buffered saline and then subjected to Western blot analysis. The samples were separated by 7.5–10% sodium sulfate‒polyacrylamide gel electrophoresis (SDS‒PAGE) and then transferred to a polyvinyl difluoride (PVDF) membrane (Merck, USA). The membranes were then analysed for protein expression by immunoblotting using the indicated antibodies. A GAPDH monoclonal antibody was used to detect the expression of GAPDH to confirm equal protein sample loading. The following antibodies were used: mouse anti-HA monoclonal antibody (1:5000 dilution), mouse anti-Flag monoclonal antibody (1:2000 dilution), mouse anti-Myc monoclonal antibody (1:2000 dilution), mouse anti-GAPDH monoclonal antibody (1:5000 dilution), mouse anti-PDCoV-N polyclonal antibody (1:1000 dilution), rabbit anti-STING polyclonal antibody (1:1000 dilution), rabbit anti-HA polyclonal antibody (1:2000 dilution), rabbit anti-Flag polyclonal antibody (1:2000 dilution), rabbit anti-Myc polyclonal antibody (1:1000 dilution), peroxidase-conjugated goat anti-mouse IgG (1:7000 dilution) and anti-rabbit IgG (1:7000 dilution).

### RNA extraction and quantitative real-time PCR

Total RNA was extracted from cultured cells using TRIzol reagent (Invitrogen, USA) and subsequently reverse-transcribed to cDNA using a HiScript III 1st Strand cDNA Synthesis Kit (Vazyme, China). Quantitative real-time PCR (RT‒qPCR) was conducted in triplicate using HiScript IV RT SuperMix for qPCR (Vazyme) on an Applied Biosystems 7500 Fast real-time PCR system (USA). Relative mRNA expression levels were normalized to the geometric mean of porcine *GAPDH*, *ACTB*, and *B2M* as reference genes and were calculated using the 2^−ΔΔCT^ method, assuming 100% efficiency of qPCR assays [33]. The qPCR primers used are listed in Table 2.

**Table 2:** **Primer sequences for RT‒qPCR**

Primers	Sequence (5′ → 3′)
pACTB-QF	GGACTTCGAGCAGGAGATGG
pACTB-QR	AGGAAGGAGGGCTGGAAGAG
pB2M-QF	ACTTTTCACACCGCTCCAGT
pB2M-QR	CGGATGGAACCCAGATACAT
pGAPDH-QF	TCTGGCAAAGTGGACATT
pGAPDH-QR	GGTGGAATCATACTGGAACA
pIFNβ-QF	ACCAACAAAGGAGCAG
pIFNβ-QR	TTTCATTCCAGCCAGT
pIFNλ1-QF	GGTGCTGGCGACTGTGATG
pIFNλ1-QR	GATTGGAACTGGCCCATGTG
pMx1-QF	AGTATGGCTCCGATATTC
pMx1-QR	CAGTTCCTCTCCTTGTAT
pISG56-QF	AATTAGCCACAGGTCATT
pISG56-QR	ATTCCATACACAACACTCT
pSTING-QF	CTGAAGTCTCTGCAATCT
pSTING-QR	ACCCGATGTAATAAGACC

### Indirect immunofluorescence assay

LLC-PK1 cells were seeded onto microscope coverslips, placed into 12-well plates, and allowed to reach approximately 60% confluence. After transfection, the cells were stained with 1 μM ER-Tracker Red (Beyotime, China) in complete DMEM for 30 min at 37 °C to visualize the endoplasmic reticulum. The cells were then fixed with 4% paraformaldehyde for 10 min, permeabilized with methyl alcohol for 15 min, and blocked with TBST containing 5% BSA for 1 h. The cells were then incubated with the appropriate primary antibodies, washed, stained with fluorescent secondary antibodies, and washed. The fluorescence images were captured using a confocal laser scanning microscope (Nikon, Japan). Pearson’s R value between the indicated proteins was quantified using ImageJ software. The following antibodies were used: rabbit anti-Flag polyclonal antibody (1:1000 dilution), mouse anti-Myc monoclonal antibody (1:1000 dilution), Alexa Fluor 488-conjugated goat anti-rabbit IgG (1:200 dilution), and Alexa Fluor 647-conjugated goat anti-mouse IgG (1:500 dilution).

### Statistical analysis

The data are expressed as the mean ± standard deviation. The normality of the distribution of the data was assessed using the Shapiro‒Wilk test. Significance was determined with two-tailed independent Student’s *t* tests and one-way analysis of variance (ANOVA). *P* values less than 0.05 were considered to indicate statistical significance (ns, not significant; *P < 0.05 and **P < 0.001).

## Results

### PDCoV nsp2 inhibits pcGAS-pSTING-induced type I and III IFN production

**Figure 1:**
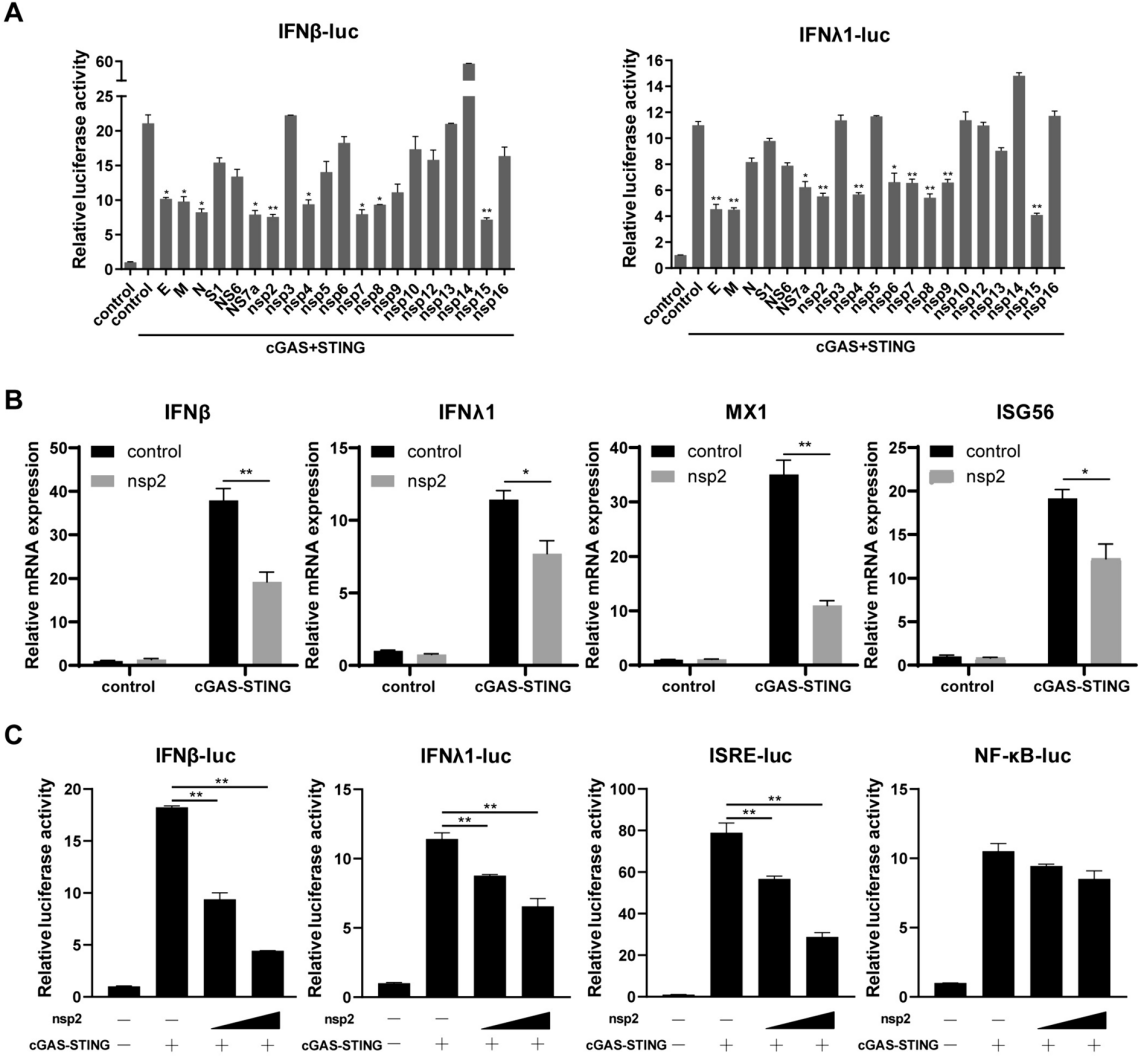
**PDCoV nsp2 inhibits pcGAS-pSTING-induced type I and III IFN production. A** LLC-PK1 cells were co-transfected with Flag-pSTING, HA-pcGAS, pRL-TK and Myc-tagged PDCoV-derived expression plasmids or an empty vector as a control, along with pGL3-pIFNβ or  pGL3-pIFNλ1. At 24 h post-transfection, the cells were lysed for dual-luciferase assays.** B** LLC-PK1 cells were co-transfected with Myc-nsp2 or an empty vector together with Flag-pSTING and HA-pcGAS for 24 h. The cells were lysed for RNA extraction. RT‒qPCR was used to detect the relative expression of pIFNβ, pIFNλ1, pMx1, and pISG56 mRNA. All gene expression levels were normalized to the geometric mean of the expression levels of the reference genes porcine *GAPDH*, *ACTB*, and *B2M*. **C** LLC-PK1 cells were co-transfected with Flag-pSTING, HA-pcGAS, pRL-TK and increasing amounts of Myc-nsp2 or an empty vector as a control, along with pGL3-pIFNβ, pGL3-pIFNλ1, pGL3-pISRE or pGL3-pNFκB. At 24 h post-transfection, the cells were lysed for dual-luciferase assays. All the data are presented as the means ± SDs of three independent experiments (*P < 0.05 and **P < 0.01).

To screen which viral proteins of PDCoV may play a role in porcine pcGAS-pSTING-induced type I and III IFN suppression, we evaluated pIFNβ and pIFNλ1 luciferase reporter activity using a series of plasmids expressing different PDCoV genes. The results showed that PDCoV E, M, NS7a, nsp2, nsp4, nsp7, nsp8, and nsp15 could significantly suppress the activation of the pIFNβ and pIFNλ1 promoters triggered by pcGAS-pSTING (Figure 1A). Given that nsp2 plays potential pleiotropic roles in the CoV lifecycle, we focused on nsp2, a nonstructural protein with functions and mechanisms that have not been well studied. To determine whether PDCoV nsp2 could inhibit the production of porcine type I and III IFNs induced by pcGAS-pSTING, RT‒qPCR was used to detect the expression of pIFNβ, pIFNλ1, and pISGs in LLC-PK1 cells. Ectopic expression of PDCoV nsp2 decreased the mRNA levels of pIFNβ, pIFNλ1, and the downstream antiviral genes pMX1 and pISG56 compared with those in control cells (Figure 1B). Furthermore, dual-luciferase reporter assays revealed that the pcGAS-pSTING-induced pIFNβ, pIFNλ1, and pISRE promoter activation was dose-dependently inhibited by PDCoV nsp2 in LLC-PK1 cells (Figure 1C). Similar results were observed in dual luciferase reporter gene assays in IPEC-J2 cells (Additional file 1). PDCoV nsp2 also significantly suppressed the activation of the pIFNβ and pIFNλ1 promoters induced by the dsDNA agonist poly(dA:dT) in LLC-PK1 cells, suggesting that nsp2 could inhibit the endogenous pcGAS-pSTING pathway (Additional file 2). These data confirmed that PDCoV nsp2 acted as an antagonist of type I and III IFNs induced by pGAS-pSTING.

### PDCoV nsp2 targets pSTING to antagonize type I and III IFNs

**Figure 2:**
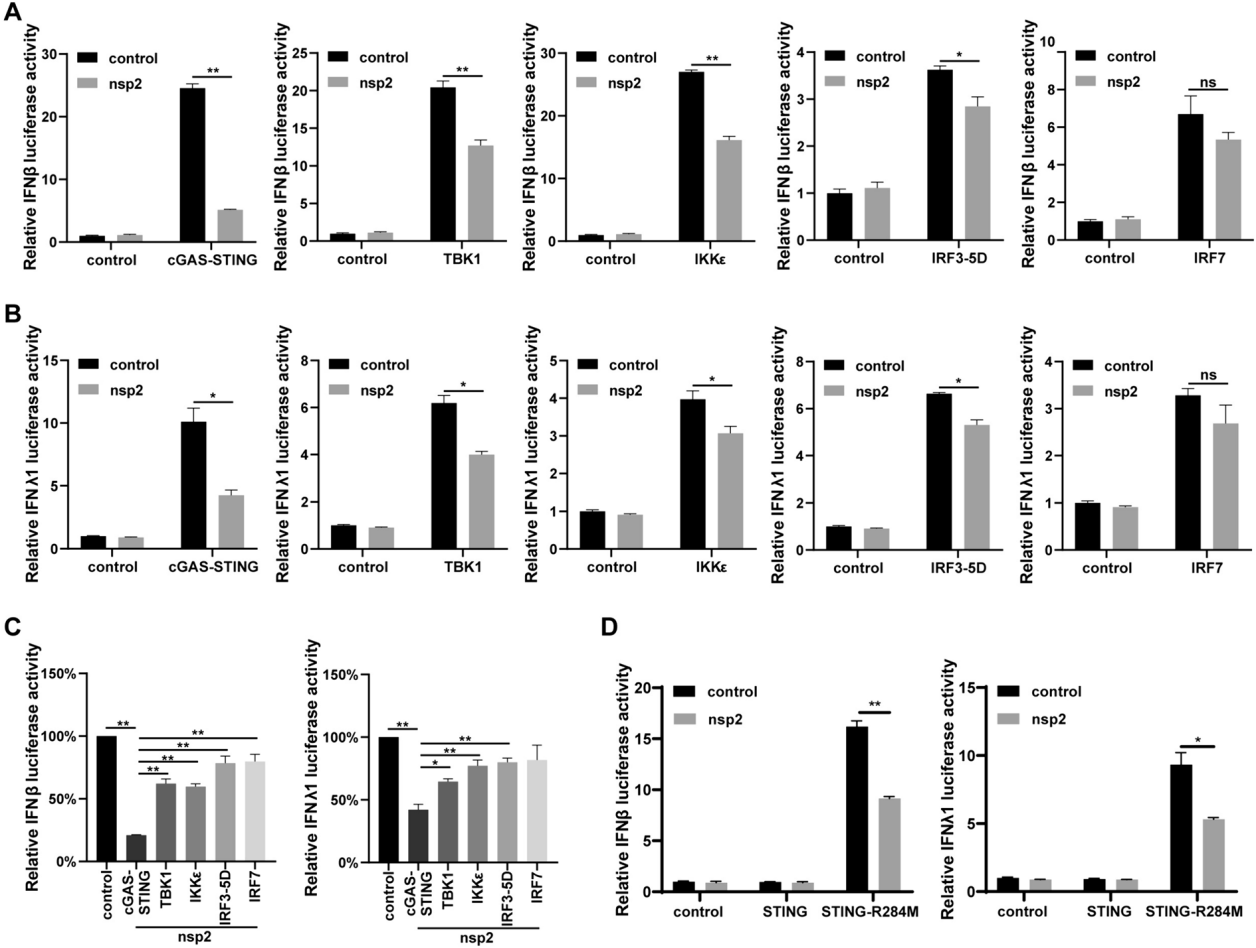
**PDCoV nsp2 targets pSTING to antagonize type I and III IFN. A, B** LLC-PK1 cells were co-transfected with Myc-nsp2 or an empty vector, and Flag-pSTING, HA-pcGAS, Flag-tagged porcine TBK1, IKKε, IRF3-5D, IRF7 or an empty vector as a control, along with pGL3-pIFNβ **A** and pGL3-pIFNλ1 **B**. At 24 h post-transfection, the cells were lysed for dual-luciferase assays. **C** The luciferase activities of pGL3-pIFNβ and pGL3-pIFNλ1 were set to 100% according to **A**, **B**. **D** LLC-PK1 cells were co-transfected with Myc-nsp2 or an empty vector and Flag-pSTING-R284M, Flag-pSTING or an empty vector as a control, along with pGL3-pIFNβ and pGL3-pIFNλ1. At 24 h post-transfection, the cells were lysed for dual-luciferase assays. All the data are presented as the means ± SDs of three independent experiments (*P < 0.05 and **P < 0.01).

To further identify the targets of PDCoV nsp2 in the pcGAS-pSTING-induced type I and III IFN signalling pathways, LLC-PK1 cells were co-transfected with nsp2 and several expression plasmids containing key molecules from the pcGAS-pSTING signalling pathway (including pcGAS-pSTING, pTBK1, pIKKε, pIRF3-5D, and pIRF7), together with the pIFNβ or pIFNλ1 luciferase reporter plasmids. We found that PDCoV nsp2 was able to suppress the activities of the pIFNβ (Figure 2A) and pIFNλ1 (Figure 2B) promoters induced by pcGAS-pSTING, pTBK1, pIKKε, and pIRF3-5D but not pIRF7. Moreover, we observed that the pIFNβ and pIFNλ1 promoter activities induced by pcGAS-pSTING were inhibited to a greater extent than those induced by downstream factors in the pcGAS-pSTING pathway, such as pTBK1, pIKKε and pIRF3-5D (Figure 2C). We wondered whether PDCoV nsp2-mediated inhibition of type I and III IFNs was primarily attributed to the suppression of pSTING. To validate this hypothesis, pSTING R284M (Arg to Met), an activation mutant of STING that is independent of cGAMP, was used in dual-luciferase reporter assays. As expected, PDCoV nsp2 was able to significantly suppress the activities of pIFNβ and pIFNλ1 induced by pSTING R284M in the absence of cGAS (Figure 2D). These findings collectively suggested that pSTING may be the target of PDCoV nsp2 to inhibit porcine type I and III IFN production.

### PDCoV nsp2 interacts with pSTING

**Figure 3:**
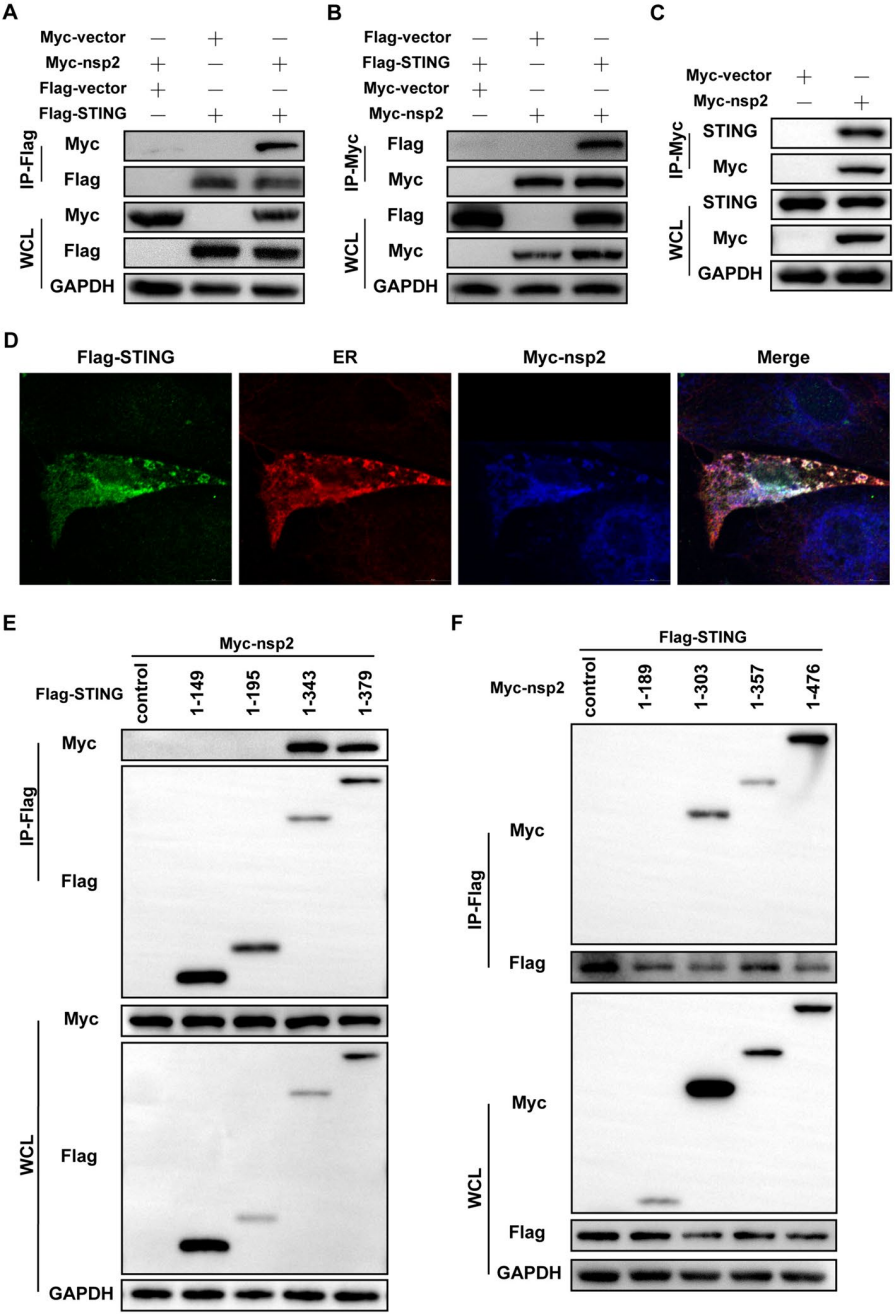
**PDCoV nsp2 interacts with pSTING. A**, **B** HEK293T cells were co-transfected with Flag-pSTING and Myc-nsp2 or the corresponding empty vector. At 28 h post-transfection, the cells were lysed for Co-IP with Flag-affinity magnetic beads or Myc-affinity magnetic beads, followed by Western blotting with anti-Flag, anti-Myc, and anti-GAPDH antibodies.** C** LLC-PK1 cells were transfected with Myc-nsp2 or an empty vector. At 28 h post-transfection, the cells were lysed for Co-IP with Myc-affinity magnetic beads and subjected to Western blotting with anti-STING, anti-Myc, and anti-GAPDH antibodies.** D** LLC-PK1 cells were co-transfected with Flag-pSTING and Myc-nsp2. At 28 h post-transfection, the cells were fixed for immunofluorescence assays to detect pSTING (green) and nsp2 (blue) with anti-Flag and anti-Myc antibodies, respectively. The ER was stained with ER-Tracker (red). Fluorescence images were acquired with a confocal laser scanning microscope (scale bar: 10 μm).** E** HEK293T cells were co-transfected with Myc-nsp2 and truncated Flag-tagged pSTING (aa 1 to 149, aa 1 to 195, aa 1 to 343, and aa 1 to 379). At 28 h post-transfection, the cells were lysed for Co-IP with Flag-affinity magnetic beads, followed by Western blotting.** F** HEK293T cells were co-transfected with Flag-pSTING and Myc-tagged truncated nsp2 (aa 1 to 189, aa 1 to 303, aa 1 to 357, and aa 1 to 476). At 28 h post-transfection, the cells were lysed for Co-IP with Flag-affinity magnetic beads, followed by immunoblotting. Western blotting was used to detect the proteins in the WCLs and immunoprecipitates with anti-Flag, anti-Myc, or anti-GAPDH antibodies.

To investigate whether nsp2 antagonized IFNs through direct interaction with STING, transient transfection and co-IP experiments were conducted. The results showed that PDCoV nsp2 was associated with pSTING in HEK293T cells (Figure 3A). Reverse co-IP also confirmed their interaction (Figure 3B). Moreover, PDCoV nsp2 could bind to endogenous pSTING in LLC-PK1 cells and IPEC-J2 cells (Figure 3C and Additional File 3). The colocalization of pSTING with nsp2 was observed in the endoplasmic reticulum (ER), with a Pearson correlation coefficient of 0.87 (Figure 3D).

To explore the domain of pSTING involved in the interaction with PDCoV nsp2, truncated mutants (aa 1 to 149, aa 1 to 195, aa 1 to 343, and aa 1 to 379) of pSTING were constructed and subjected to co-IP with PDCoV nsp2. The co-IP assay showed that the N-terminal fragment without the CTT domain of pSTING was sufficient for interaction with nsp2 (Figure 3E and Additional file 4A). The STING CTT domain is essential for IFN induction. To further identify the PDCoV nsp2 domains responsible for binding to pSTING, we constructed truncated nsp2 proteins (aa 1 to 189, aa 1 to 303, aa 1 to 357, and aa 1 to 476) and subjected them to co-IP with pSTING. We found that PDCoV nsp2 aa 1 to 303 clearly coprecipitated with pSTING, indicating that the N-terminal region of PDCoV nsp2 was responsible for the interaction (Figure 3F and Additional file 4B). Together, these results demonstrated that the N-terminal region of nsp2 interacted with the pSTING-ΔCTT domain, thereby regulating the IFN signalling pathway.

### PDCoV nsp2 promotes the degradation of pSTING

**Figure 4:**
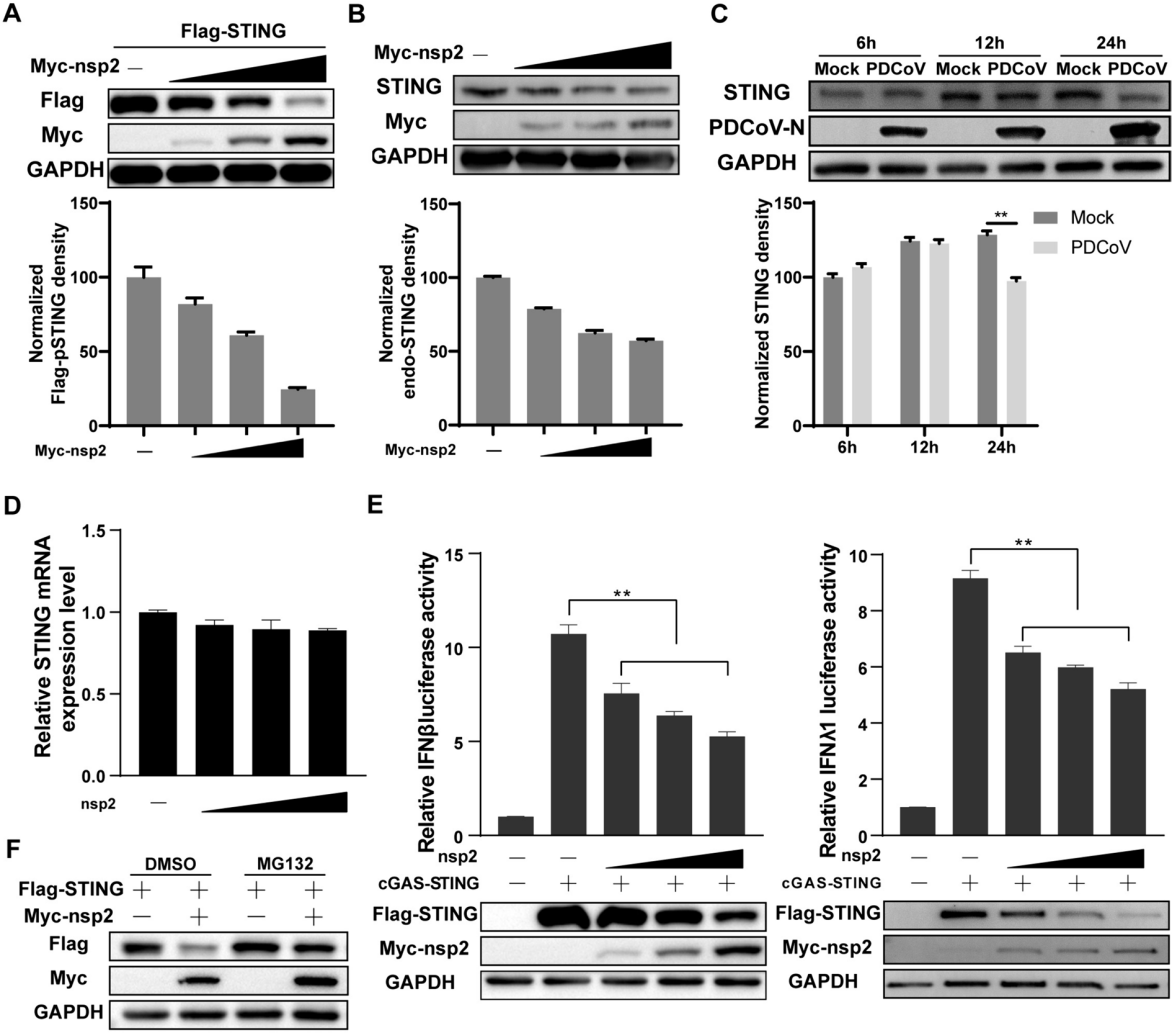
**PDCoV nsp2 promotes the degradation of pSTING A** LLC-PK1 cells were transfected with Flag-pSTING and increasing amounts of Myc-nsp2 (100 ng, 200 ng, or 500 ng) or an empty vector for 24 h, followed by Western blotting with anti-Flag, anti-Myc and anti-GAPDH antibodies. **B** LLC-PK1 cells were transfected with increasing amounts of Myc-nsp2 (100 ng, 200 ng, or 500 ng) or an empty vector. At 24 h post-transfection, the cells were lysed for Western blotting with anti-STING, anti-Myc, and anti-GAPDH antibodies. **C** LLC-PK1 cells were left uninfected or infected with PDCoV at an MOI of 1. The cells were harvested at 6 h, 12 h, or 24 h for Western blotting with anti-STING, anti-PDCoV-N, and anti-GAPDH antibodies. **D** LLC-PK1 cells were transfected with increasing amounts of Myc-nsp2 (100 ng, 200 ng, or 500 ng) or an empty vector. At 24 h post-transfection, the cells were lysed for RNA extraction. RT‒qPCR was used to detect the relative expression of pSTING mRNA. **E** LLC-PK1 cells were co-transfected with Flag-pSTING, HA-pcGAS, pRL-TK and increasing amounts of Myc-nsp2 (100 ng, 200 ng, 500 ng, or 750 ng) or an empty vector as a control, along with pGL3-pIFNβ or  pGL3-pIFNλ1. At 24 h post-transfection, the cells were lysed for dual-luciferase assays and Western blotting. **F** LLC-PK1 cells were co-transfected with Flag-pSTING and Myc-nsp2 or an empty vector. At 16 h post-transfection, the cells were treated with MG132 (20 μM) or DMSO as a control for 8 h before being harvested for Western blotting. All the experiments were performed independently three times.

STING protein expression during viral infection is closely related to its function. In this study, we also observed decreased expression of pSTING in cells expressing nsp2 (Figure 3A). To determine whether PDCoV nsp2 could affect the expression of pSTING, increasing amounts of PDCoV nsp2 were co-transfected with or without the pSTING expression plasmid. Western blot analysis showed that both the exogenous pSTING and endogenous pSTING protein expression levels could be suppressed by PDCoV nsp2 in a dose-dependent manner (Figures 4A and B). Moreover, endogenous pSTING protein expression was analysed in the context of PDCoV infection. We found that PDCoV strongly downregulated endogenous pSTING expression in infected cells (Figure 4C). However, the RT‒qPCR results revealed that PDCoV nsp2 did not affect the level of pSTING mRNA (Figure 4D). This indicated that nsp2-mediated downregulation of pSTING did not occur at the transcriptional level.

To further determine whether PDCoV nsp2 degraded pSTING during nsp2-mediated type I and III IFN production inhibition, the pcGAS and pSTING expression plasmids and increasing amounts of the PDCoV nsp2 plasmid, along with the pIFNβ or pIFNλ1 luciferase reporter plasmids, were co-transfected, followed by dual-luciferase reporter assays and Western blot analysis. As expected, the luciferase activities of pcGAS-pSTING-induced pIFNβ and pIFNλ1 were significantly inhibited in a dose-dependent manner by increasing the expression of PDCoV nsp2. Moreover, a gradual decrease in the pSTING protein level was detected (Figure 4E). Taken together, these findings indicated that PDCoV nsp2 could inhibit pcGAS-pSTING-induced type I and III IFN production by degrading pSTING. To explore the mechanisms of nsp2-mediated pSTING protein degradation, we treated nsp2-pSTING-expressing cells with the proteasome inhibitor MG132 and found that the nsp2-induced degradation of pSTING was strongly blocked by MG132 compared with that in DMSO-treated cells (Figure 4F). Collectively, these results suggested that PDCoV nsp2 promoted the degradation of pSTING via the ubiquitin‒proteasome pathway.

### PDCoV nsp2 catalyses the K48-linked ubiquitination of pSTING

**Figure 5:**
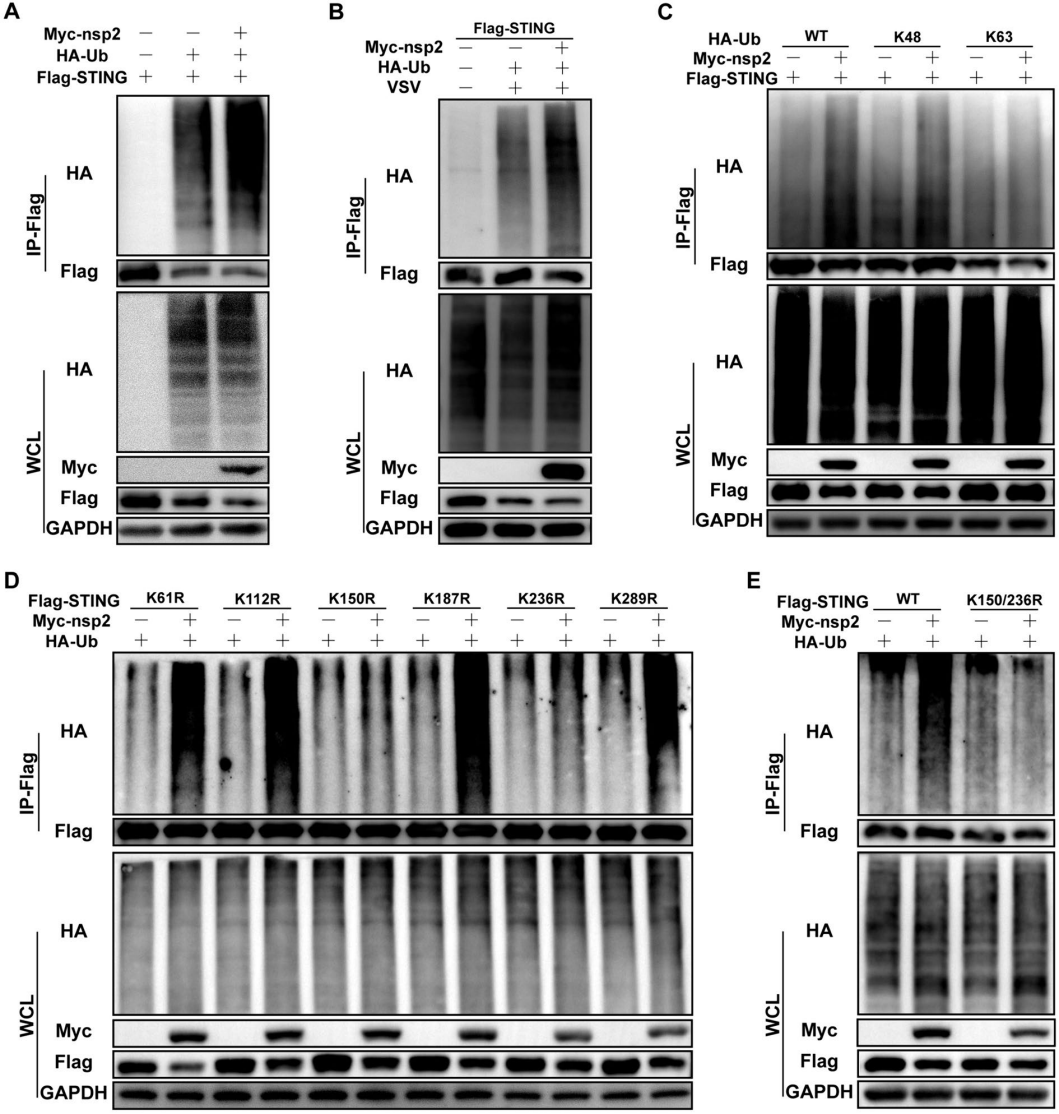
**PDCoV nsp2 catalyses K48-linked ubiquitination of pSTING. A** HEK 293 T cells were co-transfected with Flag-pSTING, Myc-nsp2 or an empty vector, and HA-Ub or an empty vector. At 24 h post-transfection, the cells were lysed for Co-IP with Flag-affinity magnetic beads and subjected to Western blotting.** B** LLC-PK1 cells were co-transfected with Flag-pSTING, Myc-nsp2 or an empty plasmid and  HA-Ub or an empty vector. At 20 h post-transfection, the cells were infected with VSV-GFP for another 8 h. The cells were lysed for Co-IP with Flag-affinity beads and subjected to Western blotting. **C** LLC-PK1 cells were co-transfected with Flag-pSTING and Myc-nsp2 or an empty vector containing HA-Ub, K48 only, or K63 only. At 28 h post-transfection, the cells were lysed for Co-IP with Flag-affinity beads and immunoblotting analysis. **D**, **E** LLC-PK1 cells were co-transfected with Myc-nsp2 or an empty vector and HA-Ub, along with the Flag-pSTING lysine mutants K61R, K112R, K150R, K187R, K236R, and K289R (**D**) or K150/236R (**E**). At 24 h post-transfection, the cells were lysed for Co-IP with Flag-affinity beads and Western blotting.

Proteosomes generally degrade proteins that are tagged with ubiquitin via proteolytic cleavage [34]. To explore whether PDCoV nsp2 regulated pSTING ubiquitination, HEK293T cells were co-transfected with ubiquitin, pSTING, and either PDCoV nsp2 or an empty vector. We found that the ubiquitination of pSTING was significantly increased by PDCoV nsp2 (Figure 5A). Moreover, PDCoV nsp2 expression dramatically increased pSTING ubiquitination after VSV-GFP infection for 8 h in LLC-PK1 cells (Figure 5B). These data demonstrated that PDCoV nsp2 promoted pSTING ubiquitination for degradation. Given that K48 and K63 chains are the most prevalent types of ubiquitin chains, we then examined the role of K48 and K63 ubiquitin chains in nsp2-mediated pSTING degradation. Immunoprecipitation analysis revealed that the ubiquitin-K48 mutant, but not the ubiquitin-K63 mutant, increased pSTING ubiquitination, indicating that PDCoV nsp2 catalysed the K48-linked ubiquitination of pSTING (Figure 5C).

Sequence analysis identified six lysine residues (K61, K112, K150, K187, K236, and K289) in pSTING. To identify the ubiquitination sites on pSTING, a series of pSTING mutants were generated by individually replacing the lysine residue with arginine, and their ubiquitination was analysed in the presence of PDCoV nsp2. K150R and K236R blocked the nsp2-mediated ubiquitination of pSTING (Figure 5D). To further identify the nsp2-mediated ubiquitination sites on pSTING, we simultaneously mutated these two lysine residues to arginines (K150/236R) and evaluated their effect on ubiquitination. The results showed that PDCoV nsp2 failed to trigger ubiquitination of the pSTING K150/236R mutant (Figure 5E). These results collectively indicated that the K150 and K236 residues of pSTING were involved in nsp2-mediated K48-linked ubiquitination of pSTING.

## Discussion

IFNs play crucial roles in the innate immune system, especially in antiviral defence [35]. Among them, type III IFN, also known as IFNλ, has attracted increasing attention in recent years due to its distinct and nonredundant functions [36]. Murine models of enteric virus infection have shown that the intestinal epithelium exclusively relies on IFNλ for protection after the neonatal period, despite all tested cell lines being responsive to IFN-α/β [37]. While transcriptome analysis has confirmed that type III IFN induces a subset of ISGs shared with type I IFN, type III IFN induces in more sustained expression of ISGs than type I IFN [38]. To establish an effective infection in the host, PDCoV has evolved multiple strategies to counteract the IFN response. To date, most studies on PDCoV evasion strategies against IFN have focused predominantly on type I IFN, which involves multiple PDCoV viral proteins, such as N, NS6, and nsp5 [24–26]. In a previous study, PDCoV infection was shown to antagonize IFN-λ1 production by reducing the peroxisome count and blocking IRF1 nuclear translocation [10]. However, the specific mechanism utilized by PDCoV still lacks a thorough exploration. We found that PDCoV infection inhibited cGAS-STING-induced pIFNβ and pIFNλ1 promoter activities in LLC-PK1 cells in our previous study (see Additional file 5). In this study, we conducted a comprehensive analysis of the impact of PDCoV proteins on both type I and III IFN production, further exploring the mechanisms underlying the regulation of IFN production by PDCoV-derived proteins. Our findings revealed that several viral proteins encoded by PDCoV, including several structural proteins and nonstructural proteins, efficiently inhibited the activation of type I and III IFN promoters induced by cGAS-STING (Figure 1A). Similar to type I IFNs, type III IFNs consist of multiple subtypes. There are four IFNs (IFNλ1, IFNλ2, IFNλ3, and IFNλ4) in humans but only three (IFNλ1, IFNλ3, and IFNλ4 but no IFNλ2) in swine [39]. Here, we showed that ectopic expression of PDCoV nsp2 suppressed the production of IFNλ1 induced by cGAS-STING (Figure 1). The basal mRNA expression levels of IFNλ3 and IFNλ4 in the tested cells were markedly lower than those of IFN-λ1. Hence, IFN-λ1 was selected exclusively as the representative type III IFN in our mechanistic investigations.

Innate cytosolic sensing of foreign nucleic acids is a key initiator of the innate immune response to viral infection [40]. Due to the PDCoV RNA genome, the RNA-sensing pathway typically plays a major role, but the interplay with the DNA-sensing pathway makes indirect activation by PDCoV equally vital [13]. Although STING serves as a well-known key adaptor molecule in the innate immune response to DNA viruses, an increasing amount of research indicates that STING evasion mechanisms are also prevalent in various RNA virus infections, including flaviviruses, influenza A virus, and coronaviruses [41]. The initial evidence of STING antagonism in RNA virus infection, as demonstrated by dengue virus (DENV), is that DENV NS2B3 can cleave human STING [42]. Additionally, the influenza virus NS1 has been shown to interact with mitochondrial DNA (mtDNA) induced by M2, inhibiting STING-dependent antiviral immunity [43]. Moreover, porcine epidemic diarrhoea virus (PEDV) and TGEV were found to deubiquitinate STING via a papain-like protease, counteracting host antiviral innate immunity by inhibiting STING activation [44, 45]. Although several studies have addressed the evasion strategies of RNA viruses against STING, it is still unclear whether PDCoV exploits similar mechanisms to evade the STING-induced IFN response. Our findings revealed for the first time that numerous viral proteins encoded by PDCoV, including structural proteins and nonstructural proteins, efficiently inhibited the pcGAS-pSTING-dependent activation of porcine IFN promoters. Furthermore, PDCoV nsp2 specifically targeted pSTING, rather than pcGAS, to evade the pcGAS-pSTING-induced IFN response.

Ubiquitination, an essential post-translational modification, is widely recognized as the most efficient regulatory mechanism of STING [46]. In the present study, we found that PDCoV nsp2-mediated degradation of pSTING was suppressed by the proteasome inhibitor MG132 (Figure 4F). Moreover, PDCoV nsp2 promoted the ubiquitination of pSTING (Figure 5A and B). These results indicated that PDCoV nsp2 promoted the degradation of pSTING through the ubiquitin‒proteasome pathway. Notably, PDCoV nsp2 did not completely lose its ability to induce degradation of pSTING, suggesting that nsp2 may utilize other cellular mechanisms to degrade pSTING. In addition to the ubiquitin‒proteasome pathway, lysosomal degradation through selective autophagy is also an important mechanism [47]. Confocal microscopy revealed that PDCoV nsp2 and pSTING were mainly colocalized in the cytoplasm with obvious speckle-like signals (Figure 3D). Hence, whether PDCoV nsp2 degrades pSTING through the autophagic lysosomal pathway or other noncanonical protein degradation pathways and what mechanisms are involved are issues that are currently under investigation in our laboratory.

The ubiquitination of STING at distinct lysine residues may regulate similar biological functions. For instance, RNF5 and RNF90 were found to degrade STING by promoting K48-linked ubiquitination at K150 [48, 49]. However, TRIM29 catalyses STING at K370 via K48-linked ubiquitination and subsequent degradation [50]. Additionally, autocrine motility factor receptor (AMFR) and TRIM32 target STING at K150 by K27-linked and K63-linked polyubiquitination, respectively [51, 52]. These two different E3 ubiquitin ligases target the same lysine residues in different types of ubiquitination. In this study, we demonstrated that PDCoV nsp2 promoted K48 polyubiquitination of STING (Figure 5C). Compared to transfection with wild-type STING, transfection with the K150/236R mutant resulted in significantly less degradation by PDCoV nsp2 (Figure 5E). The degradation of STING is commonly assisted by a specific E3 ubiquitin ligase. According to previous studies, E3 ubiquitin ligases, including RNF5, RNF90, TRIM29, and TRIM30α, are pivotal regulators of the protein stability of STING and mainly promote K48-linked polyubiquitination [48–50]. However, we confirmed that these E3 ubiquitin ligases could not be recruited by nsp2 (Additional file 6). The immediate early protein 1 (IE1) of human cytomegalovirus (HCMV) was reported to have potential E3 ubiquitin ligase activity, facilitating the ubiquitination and degradation of its substrate Hes1 [53]. Therefore, it is possible that nsp2 itself directly ubiquitinates pSTING. However, we found that nsp2 does not contain the usual motif present in E3 ubiquitin ligase family proteins. Hence, further research is necessary to identify the E3 ubiquitin ligase responsible for facilitating PDCoV nsp2-induced pSTING ubiquitination.

In summary, our current study is the first to reveal that PDCoV nsp2 acts as an IFN antagonist. Furthermore, PDCoV nsp2 interacts directly with pSTING and induces pSTING degradation through the ubiquitin‒proteasome pathway. Although PDCoV is a single-stranded RNA virus that typically antagonizes the RLR pathway for immune evasion, our findings demonstrate that PDCoV may encode viral proteins that suppress the cGAS-STING pathway, further interfering with the host innate immune response. This study provides a foundation for functional studies of the coronavirus nsp2 protein and may assist future drug design.

### Supplementary Information

**Additional file 1**. **PDCoV nsp2 inhibits cGAS-STING-induced type I and III IFN promoter activation.** IPEC-J2 cells were co-transfected with pRL-TK, Myc-nsp2 or an empty vector, HA-pcGAS, and Flag-pSTING or the empty vector, along with pGL3-pIFNβ (**A**) or pGL3-pIFNλ1 (**B**). At 24 h post-transfection, the cells were lysed for dual-luciferase assays.

**Additional file 2**. **PDCoV nsp2 inhibits poly(dA:dT)-induced type I and III IFN promoter activation.** LLC-PK1 cells were co-transfected with pRL-TK, Myc-nsp2, or an empty vector with or without poly(dA:dT), along with pGL3-pIFNβ (**A**) or pGL3-pIFNλ1 (**B**). At 24 h post-transfection, the cells were lysed for dual-luciferase assays.

**Additional file 3**. **PDCoV nsp2 interacts with pSTING.** IPEC-J2 cells were transfected with Myc-nsp2 or an empty vector. At 28 h post-transfection, the cells were lysed for Co-IP with Myc-affinity magnetic beads and subjected to Western blotting with anti-STING, anti-TBK1, anti-IRF3, anti-Myc, and anti-GAPDH antibodies.

**Additional file 4**. **PDCoV nsp2 interacts with pSTING. A** HEK293T cells were co-transfected with Myc-nsp2 and truncated Flag-tagged pSTING (aa 1 to 149, aa 1 to 195, aa 1 to 343, and aa 1 to 379). At 28 h post-transfection, the cells were lysed for Co-IP by Myc-affinity magnetic beads, followed by Western blotting.** B** HEK293T cells were co-transfected with Flag-pSTING and Myc-tagged truncated nsp2 (aa 1 to 189, aa 1 to 303, aa 1 to 357 and aa 1 to 476). At 28 h post-transfection, the cells were lysed for Co-IP by Myc-affinity magnetic beads followed by immunoblotting. Western blotting was used to detect the proteins in the WCLs and immunoprecipitates with anti-Flag, anti-Myc, or anti-GAPDH antibodies.

**Additional file 5**. **PDCoV infection inhibits cGAS-STING-induced type I and III IFN promoter activation.** LLC-PK1 cells were co-transfected with pRL-TK, HA-pcGAS, and Flag-pSTING or the empty vector, along with pGL3-pIFNβ (**A**) or pGL3-pIFNλ1 (**B**). At 12 h post-transfection, the cells were uninfected or infected with PDCoV at an MOI of 0.1. At 24 h post-infection, the cells were lysed for dual-luciferase assays.

**Additional file 6**. **PDCoV nsp2 cannot interact with RNF5, RNF90 or TRIM29.** LLC-PK1 cells were co-transfected with Myc-nsp2 or an empty vector and porcine RNF5 (**A**), RNF90 (**B**), or TRIM29 (**C**). At 28 h post-transfection, the cells were lysed for Co-IP with Myc-affinity magnetic beads and subjected to Western blotting with anti-Flag, anti-Myc and anti-GAPDH antibodies.

## Data Availability

The data analyzed during the current study are available from the corresponding author on reasonable request.
