# Supplementary figures and images for: Porcine deltacoronavirus nonstructural protein 2 inhibits type I and III IFN production by targeting STING for degradation
Source: Vet Res. 2024 Jun 17;55:79. doi: 10.1186/s13567-024-01330-w (PMC11184774; doi:10.1186/s13567-024-01330-w)

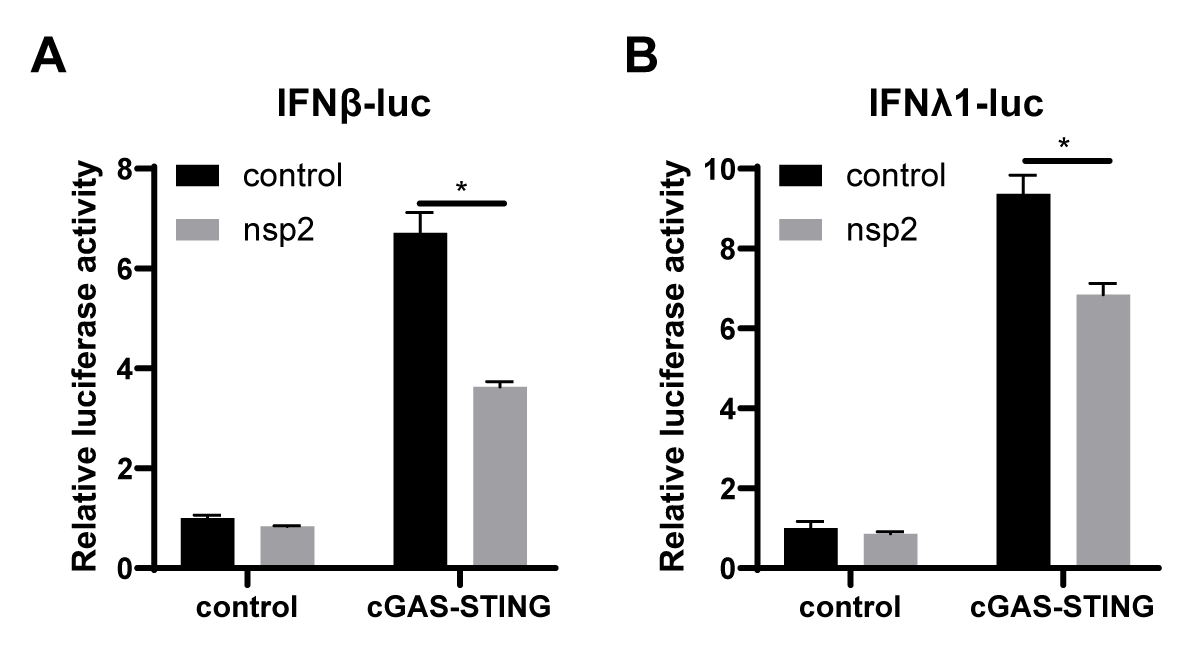

Supplement: Supplementary file 1 — Additional file 1. PDCoV nsp2 inhibits cGAS-STING-induced type I and III IFN promoter activation. IPEC-J2 cells were co-transfected with pRL-TK, Myc-nsp2 or an empty vector, HA-pcGAS, and Flag-pSTING or the empty vector, along with pGL3-pIFNβ (A) or pGL3-pIFNλ1 (B). At 24 h post-transfection, the cells were lysed for dual-luciferase assays. [file 13567_2024_1330_MOESM1_ESM.docx]

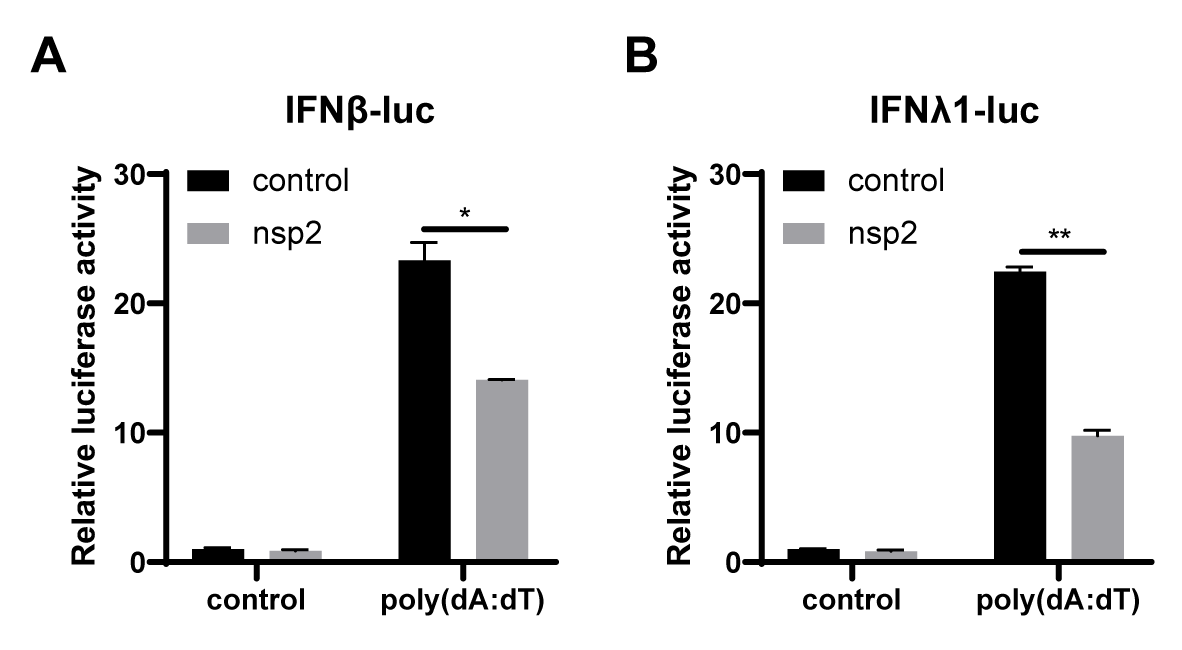

Supplement: Supplementary file 2 — Additional file 2. PDCoV nsp2 inhibits poly(dA:dT)-induced type I and III IFN promoter activation. LLC-PK1 cells were co-transfected with pRL-TK, Myc-nsp2, or an empty vector with or without poly(dA:dT), along with pGL3-pIFNβ (A) or pGL3-pIFNλ1 (B). At 24 h post-transfection, the cells were lysed for dual-luciferase assays. [file 13567_2024_1330_MOESM2_ESM.docx]

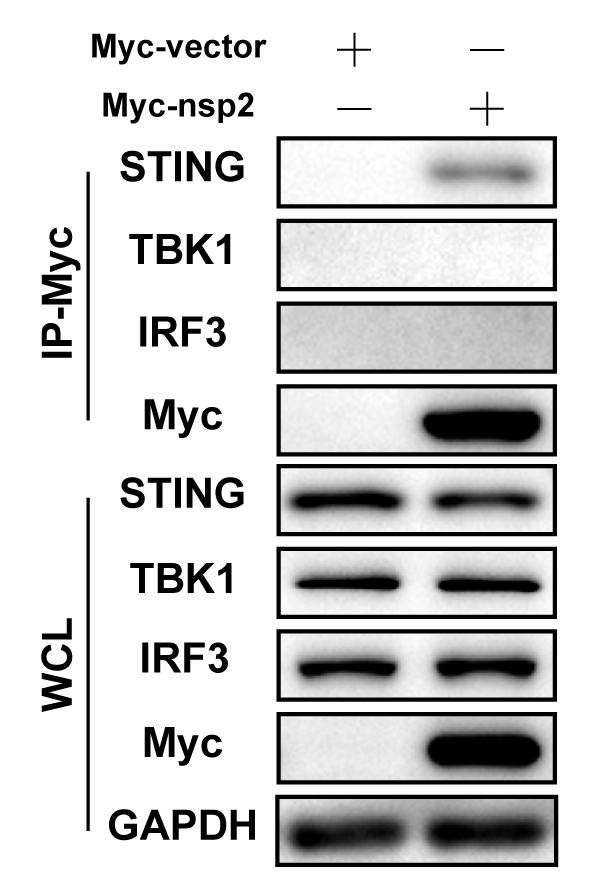

Supplement: Supplementary file 3 — Additional file 3. PDCoV nsp2 interacts with pSTING. IPEC-J2 cells were transfected with Myc-nsp2 or an empty vector. At 28 h post-transfection, the cells were lysed for Co-IP with Myc-affinity magnetic beads and subjected to Western blotting with anti-STING, anti-TBK1, anti-IRF3, anti-Myc, and anti-GAPDH antibodies. [file 13567_2024_1330_MOESM3_ESM.docx]

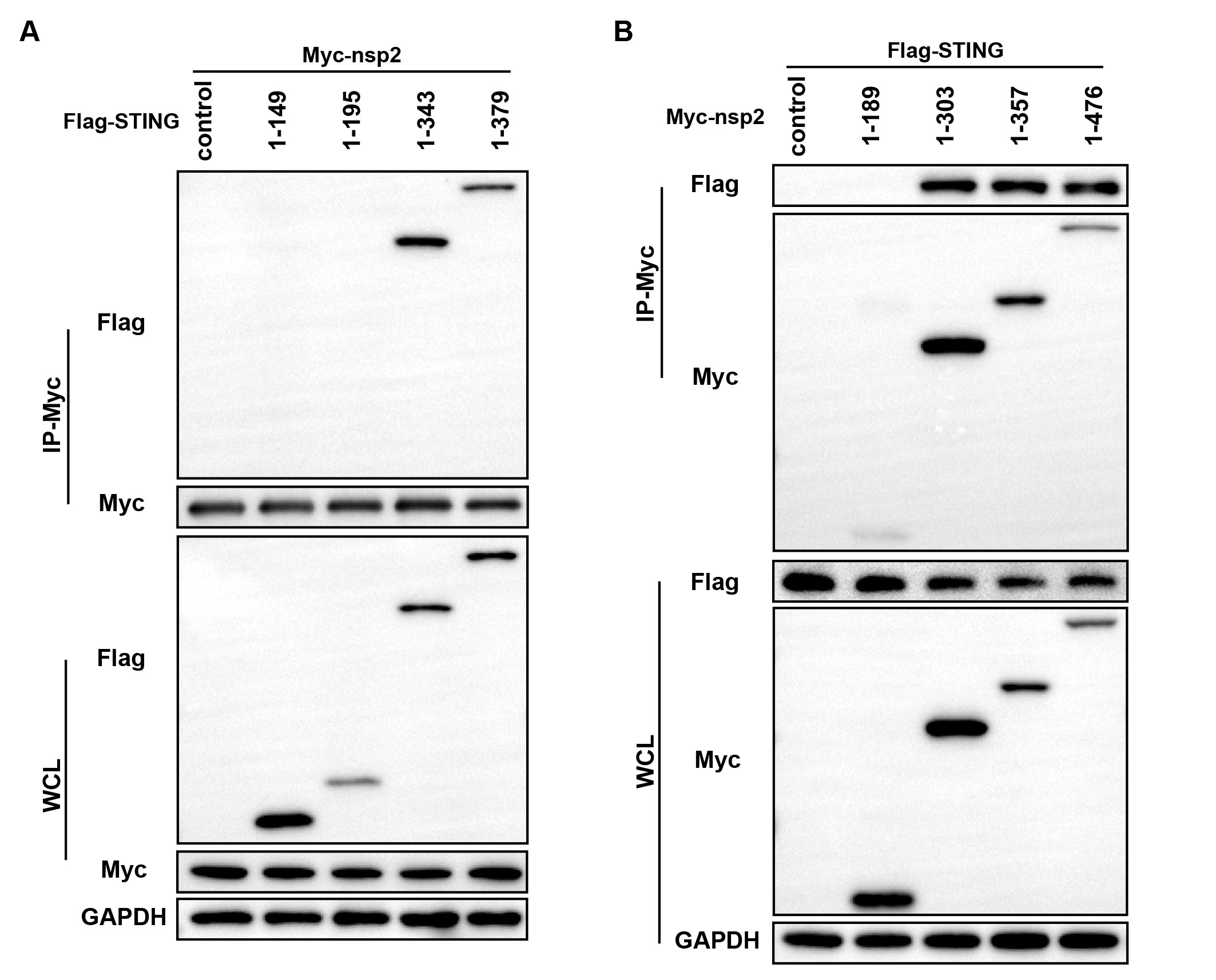

Supplement: Supplementary file 4 — Additional file 4. PDCoV nsp2 interacts with pSTING. A HEK293T cells were co-transfected with Myc-nsp2 and truncated Flag-tagged pSTING (aa 1 to 149, aa 1 to 195, aa 1 to 343, and aa 1 to 379). At 28 h post-transfection, the cells were lysed for Co-IP by Myc-affinity magnetic beads, followed by Western blotting. B HEK293T cells were co-transfected with Flag-pSTING and Myc-tagged truncated nsp2 (aa 1 to 189, aa 1 to 303, aa 1 to 357 and aa 1 to 476). At 28 h post-transfection, the cells were lysed for Co-IP by Myc-affinity magnetic beads followed by immunoblotting. Western blotting was used to detect the proteins in the WCLs and immunoprecipitates with anti-Flag, anti-Myc, or anti-GAPDH antibodies. [file 13567_2024_1330_MOESM4_ESM.docx]

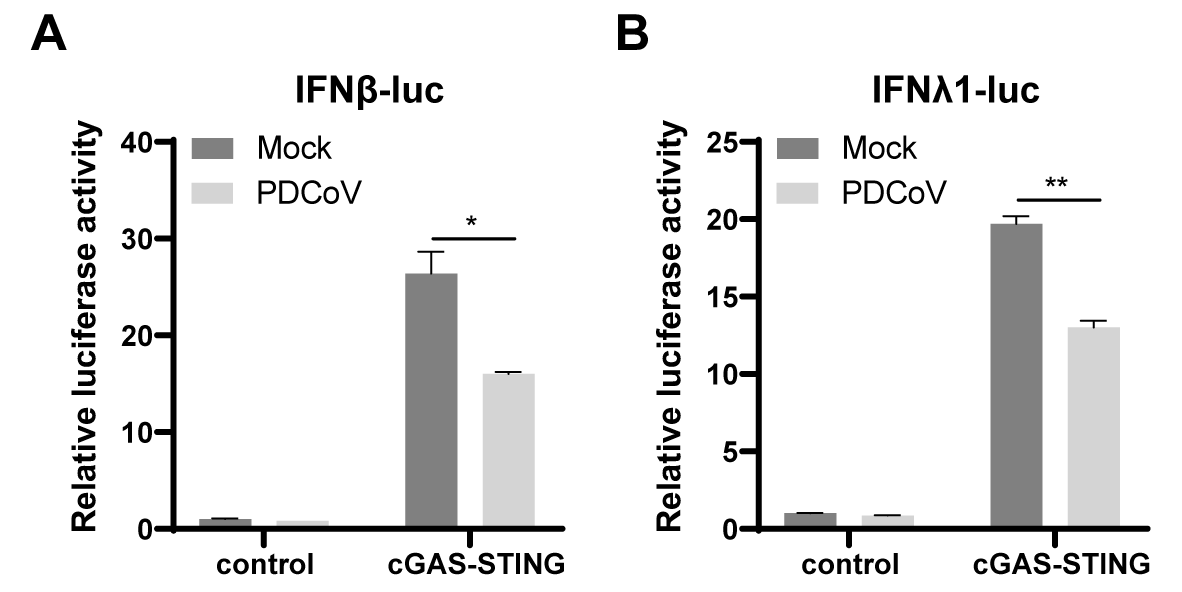

Supplement: Supplementary file 5 — Additional file 5. PDCoV infection inhibits cGAS-STING-induced type I and III IFN promoter activation. LLC-PK1 cells were co-transfected with pRL-TK, HA-pcGAS, and Flag-pSTING or the empty vector, along with pGL3-pIFNβ (A) or pGL3-pIFNλ1 (B). At 12 h post-transfection, the cells were uninfected or infected with PDCoV at an MOI of 0.1. At 24 h post-infection, the cells were lysed for dual-luciferase assays. [file 13567_2024_1330_MOESM5_ESM.docx]

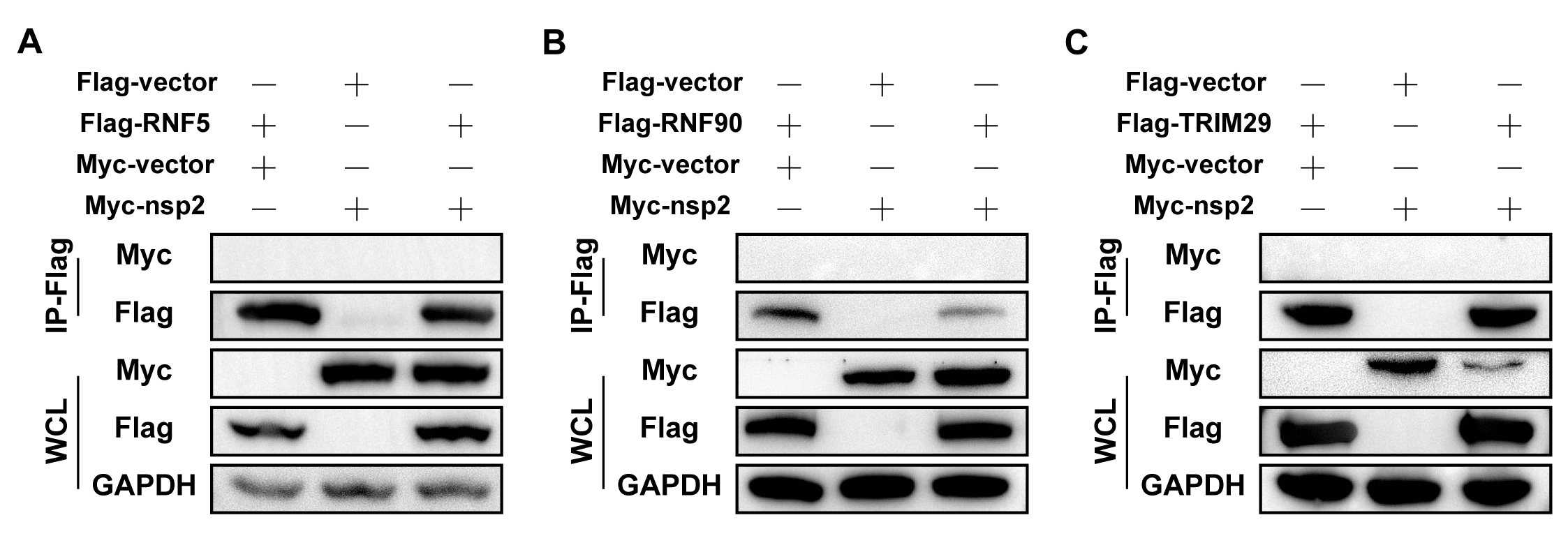

Supplement: Supplementary file 6 — Additional file 6. PDCoV nsp2 cannot interact with RNF5, RNF90 or TRIM29. LLC-PK1 cells were co-transfected with Myc-nsp2 or an empty vector and porcine RNF5 (A), RNF90 (B), or TRIM29 (C). At 28 h post-transfection, the cells were lysed for Co-IP with Myc-affinity magnetic beads and subjected to Western blotting with anti-Flag, anti-Myc and anti-GAPDH antibodies. [file 13567_2024_1330_MOESM6_ESM.docx]
